# Association of possible sarcopenia, sarcopenia and knee osteoarthritis among middle-aged and older adults: Evidence from the CHARLS cohort

**DOI:** 10.1016/j.clinsp.2026.100949

**Published:** 2026-04-17

**Authors:** Xilun Ma, Zelong Cai, Jiong Zhang, Haiqi Hu, Wanting Zheng, Ziting Peng, Haizhu Tan, Ruibin Huang

**Affiliations:** a Department of Radiology, First Affiliated Hospital of Shantou University Medical College, Shantou, Guangdong, China; b Department of Basic Medical Sciences, Shantou University Medical College, Shantou, China

**Keywords:** Knee osteoarthritis, Sarcopenia, Possible sarcopenia, CHARLS

## Abstract

**Background:**

Population-based studies exploring the association between sarcopenia, possible sarcopenia and Knee Osteoarthritis (KOA) ‒ particularly including the intermediate state of possible sarcopenia ‒ remain limited. The authors aimed to explore both cross-sectional and longitudinal associations between possible sarcopenia, sarcopenia, and KOA in a large cohort of Chinese adults.

**Methods:**

The authors utilized data from the China Health and Retirement Longitudinal Study. After applying the exclusion criteria, a total of 5018 participants aged 45-years or older were included and followed from 2011 to 2015. Sarcopenia status was classified as non-sarcopenia, possible sarcopenia, or sarcopenia according to the Asian Working Group for Sarcopenia. Logistic regression was used to assess the associations between sarcopenia status and KOA, including associations with changes in sarcopenia status over follow-up.

**Results:**

Sarcopenia and possible sarcopenia demonstrated a significant positive association with KOA, exhibiting an Odds Ratio (OR) of 1.37 (95% CI: 1.02‒1.83) and an OR of 1.55 (95% CI: 1.26‒1.91), respectively. Participants who newly developed sarcopenia had an increased likelihood of developing KOA than those who remained non-sarcopenic, with an OR of 1.72 (95% CI: 1.27‒2.32). The incidence of KOA differed significantly among the non-sarcopenia, possible sarcopenia, and sarcopenia groups, with rates of 10.0% in the non-sarcopenia group, 17.8% in the possible sarcopenia group, and 16.1% in the sarcopenia group (p*-*value < 0.01).

**Conclusions:**

Sarcopenia and possible sarcopenia were associated with the occurrence of KOA. Individuals who newly developed sarcopenia or possible sarcopenia were at a higher risk of developing KOA during the four-year follow-up.

## Highlights

• Data were derived from the China Health and Retirement Longitudinal Study (CHARLS).
• Both cross-sectional and longitudinal analyses were performed.
• Sarcopenia and possible sarcopenia were positively associated with KOA prevalence.
• Participants developing sarcopenia during follow-up had a higher risk of KOA.

## Introduction

According to data from the World Health Organization, the global population aged 60-years and older was approximately 1 billion in 2020 and is projected to reach 2.1 billion by 2050.^1^ China is experiencing a similar trend, with a marked increase in the proportion of older adults, showing that the country is becoming an aging society. Based on the latest data from the Seventh National Population Census in 2020, China had a total of 264,018,766 people aged 60 and above, accounting for 18.70 % of the total population.^2^ With the progression of aging, the most notable change in health-related behaviors among older adults is a decline in physical function. Knee Osteoarthritis (KOA) is a common degenerative arthropathy in older adults, mainly manifested by knee pain and mobility disorders. In 2020, the global age-standardized prevalence of KOA was 4307.4 cases per 100,000 population.^3^ Consistent with this high global burden, a nationwide survey in China reported a prevalence of 8.1 % among adults aged over 45-years.^4^ Severe KOA requires expensive joint replacement surgery, thereby imposing a substantial burden on families and society.^5^ Consequently, early detection and intervention of KOA are of great clinical importance. It is widely acknowledged that joints and muscles, which are integral components of the musculoskeletal system, undergo a gradual decline in function as individuals age. The interplay between these tissues plays a crucial role in preserving musculoskeletal health. Recent studies indicate that muscle atrophy may contribute to the onset and progression of KOA.^6^, ^7^, ^8^ The decline in muscle strength leads to decreased joint stability, ultimately hastening the deterioration of joint cartilage.^7^^,^^8^ Sarcopenia is a skeletal muscle disease associated with aging, characterized by loss of muscle mass, decreased muscle strength, and decline in physical function among older individuals.^9^ Recently, the association between sarcopenia and KOA has drawn growing attention, highlighting the need for further investigation into their underlying mechanisms and clinical implications.

In recent years, several studies have investigated this association; however, the results are controversial.^10^, ^11^, ^12^, ^13^, ^14^, ^15^, ^16^, ^17^ Veronese et al. discovered that the existence of sarcopenia at the baseline was significantly related to a higher risk of KOA in the research involving 2492 North American older adults, over a follow-up period of four years.^11^ Andrews et al. demonstrated that appendicular lean mass and handgrip strength, which are indicators of sarcopenia, might be associated with the development of KOA in a study including 2779 subjects from the Health, Aging, and Body Composition Study.^12^ However, a study by Ruhdorfer et al., involving 55 participants from the Osteoarthritis Initiative, showed no significant association between thigh muscle cross-sectional area derived by MRI and KOA.^15^ Similarly, Misra et al. reported that sarcopenia was not associated with KOA in a large longitudinal cohort consisting of 3026 subjects.^16^ Nevertheless, the existing studies have several limitations. Firstly, previous studies were predominantly cross-sectional surveys, limiting their ability to establish causal relationships. Secondly, the indicators utilized for evaluating sarcopenia were relatively limited, i.e., using only thigh muscle cross-sectional area or handgrip strength. These metrics alone cannot provide a comprehensive evaluation of both muscle mass and function. Lastly, according to the Asian Working Group 2019 (AWGS 2019), sarcopenia was categorized into possible sarcopenia, sarcopenia, and severe sarcopenia.^9^ However, previous studies mainly focused on the relationship between sarcopenia and KOA. To the best of our knowledge, no study has been carried out on the relationship between possible sarcopenia and KOA. The AWGS 2019 utilized the concept of possible sarcopenia to identify and intervene at the early stages of sarcopenia.^9^ Therefore, the investigation into the correlation between possible sarcopenia and KOA holds greater significance for early intervention.

China Health and Retirement Longitudinal Study (CHARLS) is a longitudinal study collecting high-quality data on Chinese individuals aged 45 and above to analyze population aging, with its national baseline survey initiated in 2011. In addition, CHARLS contains a comprehensive dataset covering multiple dimensions of sarcopenia, including physical performance, muscle strength, and Appendicular Skeletal Muscle Mass (ASM). Therefore, the aim of the present study was to explore the potential dynamic relationship between sarcopenia, possible sarcopenia, and KOA based on CHARLS, to clarify whether early-stage sarcopenia is a risk factor for KOA.

## Methods

### Study population

CHARLS is a well-designed longitudinal cohort study that collects high-quality, nationally representative microdata on Chinese adults aged 45-years and older, covering multiple aspects of households and individuals. The purpose is to analyze the problem of population aging in China. The CHARLS national baseline survey was conducted in 2011, covering 150 counties, 450 villages, and 17,000 people in about 10,000 households. These samples were then followed every two to three years. Data were collected using a standardized questionnaire that assessed lifestyle, sociodemographic factors, and health-related information. Physical measurements were conducted at each 2-year follow-up, while blood samples were collected once during every two follow-up periods. The study design of CHARLS has been described in detail in previous publications.^18^ The CHARLS datasets can be accessed free of charge on the official website (http://charls.pku.edu.cn/en). The CHARLS project was approved by the Biomedical Ethics Review Committee of Peking University (IRB00001052-11015), and written informed consent was obtained from all the participants. The present study was designed and reported in accordance with the Strengthening the Reporting of Observational Studies in Epidemiology (STROBE) guidelines.

**Fig. 1:**
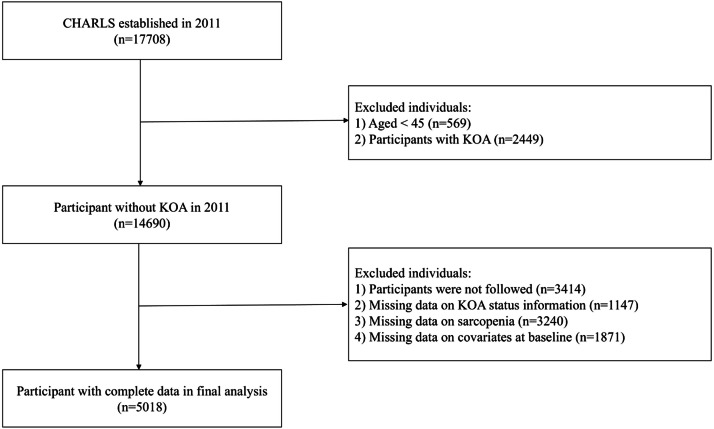
Flow of participants in the study.

A retrospective analysis was conducted on the CHARLS data from 2011 to 2015 in the present study. Inclusion criteria followed the CHARLS design: community (household) residents aged ≥45-years and their spouses. Participants were excluded based on the following criteria defined specifically for the present study: ⅰ) Aged <45-years at baseline, as CHARLS includes spouses of age-eligible participants who may be younger than 45-years; ⅱ) History of KOA or missing KOA data at baseline; ⅲ) Insufficient data to assess possible sarcopenia or sarcopenia at baseline or during follow-up; ⅳ) Missing data on KOA at follow-up; v) Participants who died or were lost to follow-up during the follow-up period; ⅵ) Missing covariates data. Ultimately, the present analysis encompassed three consecutive waves of data, comprising a comprehensive sample of 5018 individuals (Fig. 1). To evaluate potential selection bias, the authors compared baseline characteristics between participants included in the final analysis and those excluded due to missing data or loss to follow-up. The results are presented in Supplementary Table S1. Within each wave, questionnaire-based information and objective measurements were collected during the same survey visit.

### Outcomes

In this study, the primary outcome was the manifestation of symptomatic KOA, and the authors selectively included participants who, at the baseline assessment, were devoid of any reported symptomatic knee OA. The assessment of knee joint pain was conducted through the administration of a specific query: “Are you often troubled with any pains?” If the participant responded positively to this inquiry, a subsequent clarifying question was posed: “On what part of your body do you feel pain? Please list all parts of the body you are currently feeling pain”. It should be highlighted that symptomatic KOA in this study was defined based on self-reported symptoms of knee pain, and no radiological imaging or clinical diagnostic confirmation of KOA was performed.

### Measurement of sarcopenia status

Sarcopenia was assessed in accordance with the flowchart outlined in the 2019 revised guidelines by the AWGS 2019 for Sarcopenia.^9^ The assessment of sarcopenia encompassed evaluations of three aspects: physical performance, muscle strength, and ASM.

In the CHARLS study, the physical performance and muscle strength assessments were conducted by trained interviewers, who carried portable examination equipment and administered the tests in participants’ households. Handgrip strength was quantitatively assessed using a mechanical dynamometer (Yuejian™ WL-1000, Nantong, China). Participants were tested in a standing position with the elbow flexed at approximately 90°, and were guided to exert their maximum effort for a few seconds while squeezing the device. Each hand was tested twice, with the measurements performed alternately between the two hands. From the two sets of measurements obtained for each hand, the highest recorded value was selected for analysis. If handgrip strength data were available for only one hand, the measurement would be from that specific side. The criterion for low muscle strength was defined as a handgrip strength of less than 28 kilograms for men and less than 18 kilograms for women.^9^

Gait speed was evaluated by recording the participants' usual walking speed (in meters per second) over a standardized 2.5-meter course. A clean, carpet-free area of approximately 2.5 meters in length was designated as the walking course. According to the protocol, participants traversed this 2.5-meter course at their normal walking pace, with the interviewer walking alongside to ensure safety. The interviewer started timing when the participant began to walk and stopped the stopwatch when the participant reached the end of the course. The stopwatch was then reset, and the participant walked back the same distance. Then the average of these two measurements was calculated to obtain a reliable gait speed value.

The chair stand test was conducted using a standard chair with a seat height of 47 cm. Participants were asked to complete five consecutive stands from a seated position, with their arms crossed over their chest, without pausing. The outcome was the time in seconds required to complete five rises from a seated position. For analytical purposes, those who attempted but were unable to perform the full chair stand test were categorized as having low physical performance levels.

According to AWGS 2019, gait speed below 1.0 m/s or a chair stand test time of 12 seconds or more is defined as low physical performance.^9^

The estimation of muscle mass was derived from the ASM through a previously validated anthropometric formula, tailored specifically for the Chinese population:[19] ASM=0.193*weight(kg)+0.107*height(cm)−4.157*gender−0.037*age(years)−2.631.

Gender was coded as 1 for men and 2 for women. A strong agreement between X-Ray absorptiometry and the ASM equation model was observed.^19^^,^^20^ The height-Adjusted Muscle Mass (ASM/height^2^) was derived by dividing the ASM by the square of height in meters. Consistent with previous studies,^21^^,^^22^ the threshold for identifying low muscle mass was set at the 20th percentile of the ASM/height^2^ distribution within the study cohort. Thus, an ASM/height^2^ measurement below 5.29 kg/m^2^ for women and below 7.02 kg/m^2^ for men is classified as indicative of low muscle mass in Wave 1. In addition, an ASM/height^2^ measurement below 5.27 kg/m^2^ for women and below 7.00 kg/m^2^ for men is defined as low muscle mass in Wave 3.

Sarcopenia was identified as both low muscle mass and reduced physical performance or low muscle strength. Possible sarcopenia was characterized by reductions in muscle strength with or without reduced physical performance. Participants who did not meet these criteria were classified as non-sarcopenic.

After 4-years of follow-up, the non-sarcopenia group was further categorized based on changes in sarcopenia status over time into two groups: non-to-non sarcopenia group (*n* = 2464), non-to-sarcopenia group (*n* = 547). The “non-to-non sarcopenia group” referred to participants who did not develop sarcopenia from baseline to the end of the follow-up period. The “non-to-sarcopenia group” referred to participants who had no sarcopenia at baseline but developed either possible sarcopenia or sarcopenia after 4-years of follow-up.

### Covariates

At baseline, demographic and social information was gathered, including gender, age, education level (illiterate, primary school, middle school, high school/vocational high school or above), and residential area (urban, rural). Common co-morbidities were documented, including cancer, chronic lung disease, heart disease, stroke, emotional and mental disorders, arthritis, dyslipidemia, hepatic disease, kidney disease, digestive system disease, asthma, memory-related disease, hypertension, and hyperglycemia. Health-related factors include Body Mass Index (BMI), ever/current smoking, and ever/current drinking. The results of the laboratory analyses encompassed the measurement of White Blood Cell (WBC), Platelet (PLT), Glucose (GLU), Triglyceride (TG), C-Reactive Protein (CRP), Cholesterol (CHO), High-Density Lipoprotein (HDL), and Hemoglobin (HGB).

### Statistical analysis

Data were presented as median and Interquartile Range (IQR) for continuous variables and percentages for categorical variables. Categorical variables for sociodemographic, health status, and health-related characteristics were compared using the Chi-Square test, after verifying that expected cell-count assumptions were met. The normality of continuous variables was assessed using both graphical (Q-Q plots) and numerical (Skewness/Kurtosis statistics) methods. As the distributions of these variables deviated from a normal distribution, the Kruskal-Wallis Test was utilized to assess the statistical significance of differences between groups. Inter-group pairwise comparisons were performed using the Wilcoxon rank-sum test, and a Bonferroni correction was subsequently applied to account for multiple comparisons. The prevalence of KOA across various cohorts was also assessed utilizing the Chi-Square test.

Logistic regression models were used to calculate Odds Ratios (ORs) and 95 % Confidence Intervals (95 % CIs) to investigate the association between the prevalence of KOA at the 4-year follow-up and sarcopenia status at baseline. Furthermore, the authors also used logistic regression models to investigate the association between the dynamic changes of sarcopenia and the prevalence of KOA. Three models were utilized with different combinations of covariates to account for potential confounding factors. Model 1 was unadjusted. Model 2 was adjusted for age, gender, rural, smoking, and drinking statuses. Model 3 was adjusted similarly to Model 2 with further adjustment for co-morbidities (dyslipidemia, cancer, lung disease, kidney disease, hepatic disease, digestive disease, asthma, emotional and mental disorders, memory-related disease, arthritis, hypertension, hyperglycemia, heart disease, and stroke), metabolic biomarkers (WBC, GLU, TG, CRP, CHO, and HDL). Rural residence was coded as a binary variable, with urban residence serving as the reference category.

Model fit was evaluated using Akaike’s Information Criterion (AIC) and Bayesian Information Criterion (BIC). To formally compare nested models, the authors further conducted likelihood ratio tests, with statistical significance defined as a p-value <0.05.

All analyses were performed using STATA 18.0. A p-value <0.05 was considered statistically significant.

## Result

### Characteristics of participants in the baseline

All chi-square test assumptions were satisfied (minimum expected count ≥ 5). Table 1 presents the baseline characteristics of the study participants in Wave 1. The median age of the 5018 participants was 58 (IQR 52‒64) years, and 51.7 % of the participants were women. Among the participants, the prevalence rate of possible sarcopenia and sarcopenia was 22.6 % (1133/5018) and 17.4 % (874/5018), respectively, according to the suggested diagnostic algorithm of AWGS 2019.^9^ Compared to the non-sarcopenia group, the participants with sarcopenia were more likely to be older (median age, 65 vs. 56 years) and women (58.1 % vs. 47.2 %), live in rural areas (76.1 % vs. 64.3 %), and had a lower level of education (illiterate, 62.9 % vs. 37.1 %). Except for cancer, emotional and mental disorders, hepatic disease and kidney disease, the distributions of co-morbidities among non-sarcopenia, possible sarcopenia, and sarcopenia groups were observed with significant differences (all p*-*value < 0.05). A lower level of WBC was also observed in the participants with sarcopenia compared to those with possible sarcopenia (p-value < 0.05).

**Table 1:** Baseline characteristics of the participants according to sarcopenia status.

Variables	Total (5018)	Non-sarcopenia (3011)	Possible sarcopenia (1133)	Sarcopenia (874)	p-value
Age, years, median (IQR)	58 (52‒64)	56 (49‒62)	59 (54‒65)	65 (60‒72)	<0.001
Gender, n ( %)					<0.001
Male	2424 (48.3)	1590 (52.8)	468 (41.3)	366 (41.9)	
Female	2594 (51.7)	1421 (47.2)	665 (58.7)	508 (58.1)	
Residential area, n ( %)					<0.001
Urban	1663 (33.1)	1074 (35.7)	380 (33.5)	209 (23.9)	
Rural	3355 (66.9)	1937 (64.3)	753 (66.5)	665 (76.1)	
Education, n ( %)					<0.001
Illiterate	2275 (45.3)	1116 (37.1)	609 (53.8)	550 (62.9)	
Primary school	1171 (23.3)	723 (24.0)	250 (22.1)	198 (22.7)	
Middle school	1057 (21.1)	769 (25.5)	194 (17.1)	94 (10.8)	
High school/Vocational high school or above	515 (10.3)	403 (13.4)	80 (7.1)	32 (3.7)	
Ever/current smoke, n ( %)	1985 (39.6)	1249 (41.5)	402 (35.5)	334 (38.2)	0.001
Ever/current drink, n ( %)	2096 (41.8)	1366 (45.4)	421 (37.2)	309 (35.4)	<0.001
Co-morbidities, n ( %)					
Cancer	35 (0.7)	23 (0.8)	9 (0.8)	3 (0.3)	0.381
Chronic lung disease	425 (8.5)	217 (7.2)	93 (8.2)	115 (13.2)	<0.001
Heart disease	529 (10.5)	283 (9.4)	169 (14.9)	77 (8.8)	<0.001
Stroke	92 (1.8)	39 (1.3)	34 (3.0)	19 (2.2)	0.001
Emotional and mental disorders	45 (0.9)	24 (0.8)	14 (1.2)	7 (0.8)	0.388
Arthritis	1424 (28.4)	777 (25.8)	375 (33.1)	272 (31.1)	<0.001
Dyslipidemia	463 (9.2)	302 (10.0)	134 (11.8)	27 (3.1)	<0.001
Hepatic disease	146 (2.9)	84 (2.8)	37 (3.3)	25 (2.9)	0.716
Kidney disease	246 (4.9)	148 (4.9)	57 (5.0)	41 (4.7)	0.939
Digestive system disease	1045 (20.8)	564 (18.7)	258 (22.8)	223 (25.5)	<0.001
Asthma	218 (4.3)	105 (3.5)	53 (4.7)	60 (6.9)	<0.001
Memory-related disease	44 (0.9)	17 (0.6)	16 (1.4)	11 (1.3)	0.014
Hypertension	2336 (13.4)	1381 (45.9)	583 (51.5)	372 (42.6)	<0.001
Hyperglycemia	671 (46.6)	425 (14.1)	171 (15.1)	75 (8.6)	<0.001
BMI (kg/m^2^)	23.1 (20.9‒25.7)	23.8 (22.0‒26.2)	23.9 (22.2‒26.6)	19.2 (18.2‒20.1)	<0.001
Wbc (1000)	5.9 (4.9‒7.2)	6.0 (5.0‒7.2)	6.0 (4.9‒7.3)	5.8 (4.7‒6.9)	0.011
Plt (10^9^/L)	207 (162‒255)	208 (164‒256)	205 (157‒254)	205 (159‒254)	0.175
GLU (mg/dL)	102.2 (94.3‒112.9)	102.6 (94.9‒113.8)	102.6 (94.5‒113.9)	100.7 (92.9‒109.6)	<0.001
TG (mg/dL)	103.5 (74.3‒151.3)	107.1(75.2‒160.2)	111.5 (80.5‒161.1)	86.7 (65.5‒119.5)	<0.001
CRP (mg/L)	1.0 (0.5‒2.0)	1.0 (0.6‒2.0)	1.1 (0.6‒2.1)	0.8 (0.4‒1.8)	<0.001
CHO (mg/dl)	190.8 (167.0‒215.3)	190.6 (167.4‒214.9)	191.0 (167.4‒216.5)	191.6 (166.6‒216.1)	0.853
HDL (mg/dL)	49.1 (40.2‒59.9)	47.9 (39.4‒58.4)	47.2 (39.4‒57.6)	56.8 (47.6‒68.0)	<0.001
HGB (g/dL)	14.2 (13.1‒15.6)	14.5 (13.2‒15.8)	14.2 (13.1‒15.6)	13.6 (12.6‒14.8)	<0.001

Note: Median (IQR) was used for continuous variables, and the p-value was calculated using the Kruskal-Wallis Test. Numbers (percentages) were used for categorical variables, and the p-value was calculated using the Chi-Square test.

BMI, Body Mass Index; IQR, Interquartile Range; WBC, White Blood Cell; PLT, Platelet; GLU, Glucose; TG, Triglyceride; CRP, C-Reactive Protein; CHO, Cholesterol; HDL, High-Density Lipoprotein; HGB, Hemoglobin.

### Cross-sectional associations of possible sarcopenia, sarcopenia with KOA

**Fig. 2:**
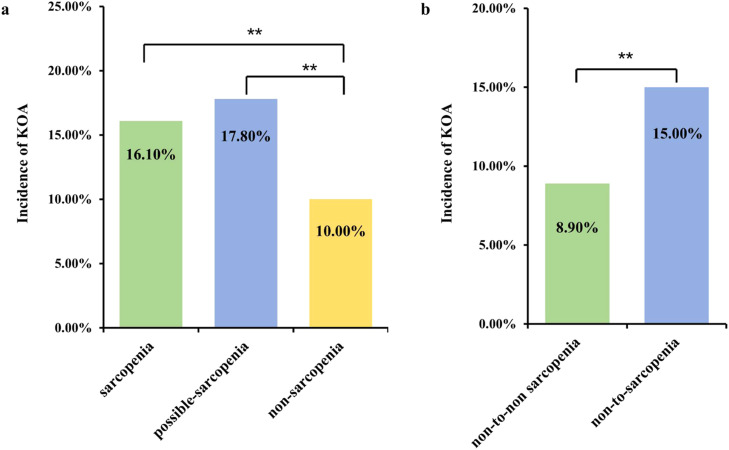
Incidence of KOA according to (a) Sarcopenia status at baseline, (b) Change in sarcopenia or possible sarcopenia status over time. ** Indicates p-value <0.01 (Chi-Square test).

**Fig. 3:**
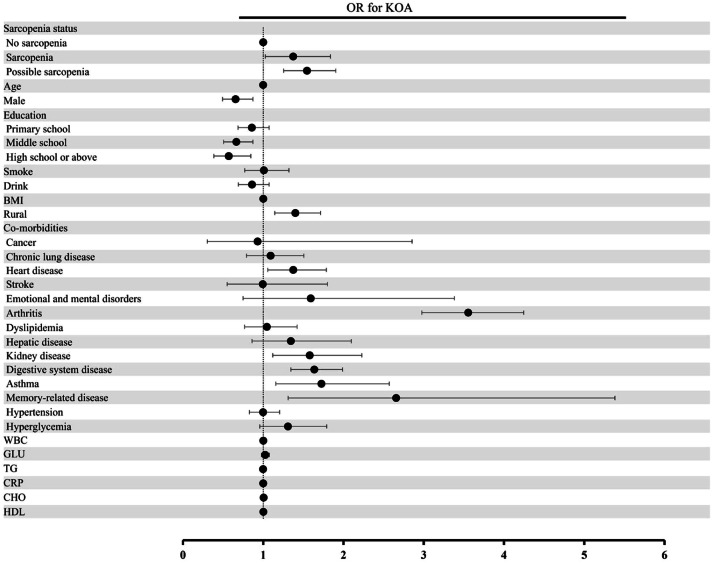
ORs and 95 % CIs of KOA by sarcopenia status in the cross-sectional analysis. Forest plot shows ORs and 95 % CIs adjusted for age, gender, rural, smoking and drinking statuses, BMI, dyslipidemia, cancer, lung disease, kidney disease, hepatic disease, digestive disease, asthma, emotional and mental disorders, memory-related disease, arthritis, hypertension, hyperglycemia, heart disease, stroke, WBC, GLU, TG, CRP, CHO and HDL. OR, Odds Ratio; CIs, Confidence Intervals; BMI, Body Mass Index; WBC, White Blood Cell; GLU, Glucose; TG, Triglyceride; CRP, C-Reactive Protein; CHO, Cholesterol; HDL, High-Density Jipoprotein.

Among the 5018 participants included in the cross-sectional study, 645 individuals (12.9 %) developed KOA at Wave 3. The incidence of KOA was significantly lower in the non-sarcopenia group than in the possible sarcopenia and sarcopenia groups, with rates of 10.0 % in the non-sarcopenia group, 17.8 % in the possible sarcopenia group, and 16.1 % in the sarcopenia group (p*-*value <0.001, Fig. 2a). However, there was no significant difference (p-value = 0.317) in the incidence of KOA between individuals with possible sarcopenia and those with sarcopenia. In Model 1, participants with sarcopenia or possible sarcopenia at baseline had a higher risk of developing KOA after 4-years of follow-up compared to baseline participants without sarcopenia, with OR of 1.73 (95 % CI 1.39‒2.14) and OR of 1.95 (95 % CI 1.61‒2.36), respectively. These associations persisted after further adjusting for other covariates (Model 2 and 3), detailed in Table 2 and Fig. 3. Therefore, the evaluation of sarcopenia status may assist in identifying populations at higher risk of developing KOA. Furthermore, Model 3 demonstrates improved model fit compared with simpler models. AIC decreased from 1234.5 in Model 1 to 1180.3 in Model 3, and BIC decreased from 1260.2 to 1210.9. The likelihood ratio test further supported the inclusion of additional covariates (p-value < 0.01).

**Table 2:** Association between the risk of KOA and possible sarcopenia or sarcopenia by logistic regression models.

Models	Non-sarcopenia	Possible sarcopenia		Sarcopenia	
		OR (95 % CI)	p-value	OR (95 % CI)	p-value
Model 1	Reference	1.95 (1.61, 2.36)	<0.001	1.73 (1.39, 2.14)	<0.001
Model 2	Reference	1.70 (1.39, 2.07)	<0.001	1.41 (1.06, 1.86)	0.016
Model 3	Reference	1.55 (1.25, 1.90)	<0.001	1.37 (1.03,1.84)	0.033

Model 1 was unadjusted.

Model 2 was adjusted for age, gender, rural, body mass index, education, smoking, and drinking statuses.

Model 3 was adjusted similarly to Model 2 with further adjustment for dyslipidemia, cancer, lung disease, kidney disease, hepatic disease, digestive disease, asthma, emotional and mental disorders, memory-related disease, arthritis, hypertension, hyperglycemia, heart disease, stroke, White Blood Cell, Glucose, Triglyceride, C-Reactive Protein, Cholesterol, and High-Density Lipoprotein. The Odds Ratio (OR) and 95 % Confidence Interval (95 % CI) were obtained from multivariate logistic regression analyses.

In stratified analyses, the associations appeared stronger among rural participants, whereas estimates in the urban subgroup did not reach statistical significance (Supplementary Table S3). Given the higher proportion of rural participants in the study population, the authors further examined whether the association between sarcopenia and KOA differed by residential setting by testing an interaction term. No statistically significant interaction was observed (p for interaction = 0.126). Post hoc power analysis indicated limited statistical power in the urban subgroup (21.8 % for possible sarcopenia and 8.3 % for sarcopenia). Baseline characteristics stratified by residential setting are presented in Supplementary Table S2.

### Longitudinal association between changes in sarcopenia status and incident KOA during 2011–2015

The incidence of KOA in the non-to-non sarcopenia group and non-to-sarcopenia group was 8.9 % and 15.0 %, respectively, with statistical significance (p*-*value < 0.001, Fig. 2b).

**Fig. 4:**
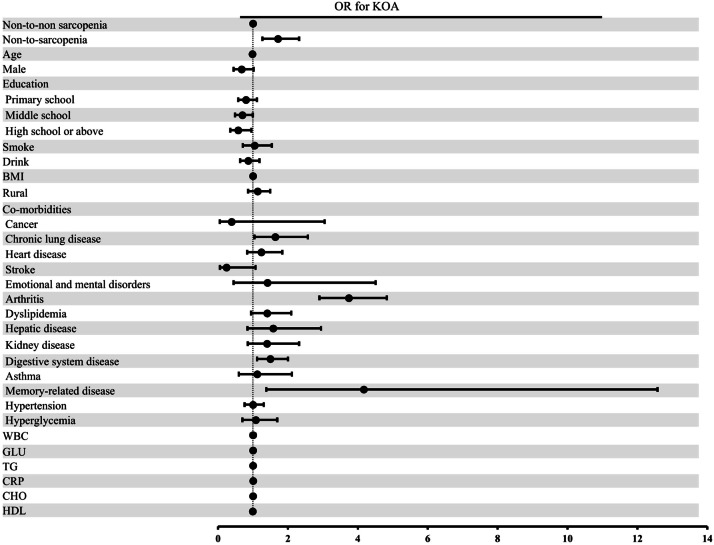
ORs and 95 % CIs of KOA by changes in sarcopenia status in the longitudinal analysis. Forest plot shows ORs and 95 % CIs adjusted for age, gender, rural, smoking and drinking statuses, BMI, dyslipidemia, cancer, lung disease, kidney disease, hepatic disease, digestive disease, asthma, emotional and mental disorders, memory-related disease, arthritis, hypertension, hyperglycemia, heart disease, stroke, WBC, GLU, TG, CRP, CHO and HDL. OR, Odds Ratio; CIs, Confidence Intervals; BMI, Body Mass Index; WBC, White Blood Cell; GLU, Glucose; TG, Triglyceride; CRP, C-Reactive Protein; CHO, Cholesterol; HDL, High-Density Lipoprotein.

In the unadjusted model (Model 1), the non-to-sarcopenia group had an increased likelihood of developing KOA than the non-to-non sarcopenia group, with an OR of 1.80 (95 % CI 1.37‒2.36), detailed in Table 3. Even after adjusting other covariates (Models 2 and 3), the result remains unchanged, with ORs of 1.74 (95 % CI 1.31‒2.30) and 1.72 (95 % CI 1.27‒2.32), respectively (Table 3 and Fig. 4). In addition, AIC values decreased from 2450.6 in Model 1 to 2385.4 in Model 3, favoring the fully adjusted model, whereas BIC favored the unadjusted specification due to its stronger penalty for complexity (2490.7 vs. 2458.9). The likelihood ratio test nonetheless indicated significant improvement with further adjustment (p-value < 0.01). Overall, the main effect estimates remained consistent across all models, underscoring the robustness of the findings.

**Table 3:** Associations between the risk of KOA and changes in possible sarcopenia or sarcopenia status during 4-years of follow-up by logistic regression models.

Models	Non-to-non sarcopenia	Non-to-sarcopenia	
		OR (95 % CI)	p-value
Model 1	Reference	1.80 (1.37, 2.36)	<0.001
Model 2	Reference	1.74 (1.31, 2.30)	<0.001
Model 3	Reference	1.72 (1.27, 2.32)	<0.001

Model 1 was unadjusted.

Model 2 was adjusted for age, gender, rural, body mass index, education, smoking, and drinking statuses.

Model 3 was adjusted similarly to Model 2 with further adjustment for dyslipidemia, cancer, lung disease, kidney disease, hepatic disease, digestive disease, asthma, emotional and mental disorders, memory-related disease, arthritis, hypertension, hyperglycemia, heart disease, stroke, White Blood Cell, Glucose, Triglyceride, C-Reactive Protein, Cholesterol, and High-Density Lipoprotein.

The Odds Ratio (OR) and 95 % Confidence Intervals (95 % CI) were obtained from multivariate logistic regression analyses.

## Discussion

This is the first longitudinal study to explore the relationship between sarcopenia, possible sarcopenia, and KOA with a large cohort of middle-aged and older Chinese adults based on the CHARLS database. The main results of this study demonstrated that individuals with sarcopenia or possible sarcopenia exhibited a significant positive correlation with the prevalence of KOA. The association remained even after accounting for multiple factors such as age, gender, rural, BMI, education level, smoking and drinking statuses, multiple chronic diseases, and metabolic biomarkers. Furthermore, participants without sarcopenia at baseline who developed sarcopenia or possible sarcopenia by the end of follow-up were at increased risk of KOA.

Several studies have suggested that sarcopenia is positively associated with KOA.^10^, ^11^, ^12^, ^13^, ^14^ For example, Kim et al. demonstrated a significant interactive correlation between sarcopenia and the presence as well as severity of KOA based on cross-sectional data, utilizing a weight-adjusted skeletal muscle index to define sarcopenia.^13^ Zhang et al. suggested a potential unidirectional causal relationship between KOA and sarcopenia, supporting the hypothesis that European individuals over the age of 60 with sarcopenia are at an increased risk of developing KOA.^10^ Although the criteria for diagnosing sarcopenia differ significantly, these findings are largely consistent with the present results. In the cross-sectional study, the authors also found that sarcopenia, as assessed by the AWGS 2019 criteria, was significantly associated with KOA. Furthermore, it suggests that dynamic changes in sarcopenia, manifested as a continuous decline in muscle mass and function, may independently increase the risk of developing KOA in the longitudinal study. Therefore, the assessment of sarcopenia status during community health checks and in routine clinical practice may help to identify populations at higher risk of developing KOA, who would benefit most from early intervention and prevention. Clinically, the dynamic assessment of sarcopenia or possible sarcopenia progression may be helpful in mitigating or delaying KOA onset among middle-aged and older adults.

Previous studies have investigated the underlying mechanisms of the relationship between sarcopenia and KOA. First, muscles contribute to joint stability by contracting and relaxing. They also provide support to the joints, distribute load evenly, and reduce excessive stress on joint cartilage. A reduction in muscle strength may lead to diminished stability of the knee joint and hasten the degeneration of articular cartilage.^23^ Second, inflammatory factors are one of the main risk factors for sarcopenia. They can also induce the onset of osteoarthritis and facilitate the progression of osteoarthritis simultaneously. According to previous studies, there is an increase in the levels of inflammatory factors such as tumor necrosis factor-alpha and interleukin-6 in patients with sarcopenia or KOA.^24^, ^25^, ^26^, ^27^, ^28^, ^29^, ^30^ Lastly, irisin, a myokine known to delay the progression of cartilage injury,^31^ has been found to be reduced in individuals diagnosed with sarcopenia,^32^ potentially increasing their susceptibility to KOA. These mechanisms were not evaluated in this study and are discussed based on prior literature.

No studies have explored the relationship between possible sarcopenia, as assessed by the AWGS 2019 criteria, and KOA. Previous research has shown that possible sarcopenia is associated with multiple aging-related diseases, highlighting its potential role as a common risk factor in the aging process.^33^, ^34^, ^35^, ^36^, ^37^ Hu et al. found that possible sarcopenia was positively associated with mild cognitive impairment among older adults in the CHARLS cohort.^33^ Meanwhile, Gao et al. demonstrated that possible sarcopenia was associated with a higher risk of cardiovascular disease among middle-aged and older Chinese adults and suggested that preventing and/or improving possible sarcopenia may be beneficial for reducing the incidence of cardiovascular disease.^34^ In addition, a few previous studies have separately demonstrated the associations between handgrip strength, walking pace, and KOA.^10^^,^^12^ A longitudinal study reported that the decrement of appendicular lean mass and handgrip strength was significantly associated with KOA among 2779 older adults.^12^ A mendelian randomization study also found that improving walking speed may reduce the risk of KOA in UK individuals.^10^ Consistent with previous studies, the present study found that possible sarcopenia was positively associated with the incidence of KOA, highlighting possible sarcopenia as a potential modifiable risk factor. Thus, maintaining enough muscle strength and/or physical performance may play an important role in the prevention of KOA among middle-aged and older adults.

The baseline findings of the present study indicated a reduced prevalence of smoking and alcohol consumption among individuals with sarcopenia or possible sarcopenia, in contrast to previous research.^17^^,^^38^ This difference may be attributed to the distinct racial and diagnostic criteria for sarcopenia between Chinese and Western populations. Alternatively, this finding could stem from reverse causality, where individuals experiencing muscle weakness or frailty may already engage in healthier behaviors. Although effect estimates appeared stronger in the rural subgroup, formal interaction testing did not support statistically significant effect modification by residential setting. The findings presented here indicate that any observed differences between the urban and rural subgroups appear to be due to differences in sample size and statistical power rather than any meaningful biological differences between the two population groups.

The present study has several strengths. First, the present study defined sarcopenia and possible sarcopenia by applying the AWGS 2019 criteria, thereby providing a more comprehensive assessment compared to previous studies. Second, a longitudinal and cross-sectional study provided a better understanding of the causal relationship between sarcopenia and KOA. Lastly, this study investigated the relationship between the dynamic changes in sarcopenia and KOA, which has not yet been reported in other articles.

There are several limitations in the present study. First, given that the CHARLS participants did not undergo radiographic assessment, the diagnosis of KOA was necessarily derived from self-reported knee pain. It is important to note that this method differs from the diagnostic criteria employed in other studies. Nevertheless, it provided an epidemiologically reasonable basis for defining KOA in this specific cohort, a precedent that has been reported in previous studies.^39^^,^^40^ Second, the information on physical activity and physical exercise had excessive missing data in CHARLS and therefore could not be incorporated as covariates in the main analysis. As low physical activity is a common risk factor for both sarcopenia and symptomatic KOA, the lack of adjustment for this covariate may have introduced residual confounding and could have led to an overestimation of the observed associations. Third, the assessment of physical performance encompassed gait speed, the 5-time chair stand test, and the short physical performance battery. Due to data limitations, the present study did not incorporate the short physical performance battery score, potentially resulting in a slight margin of error in the diagnosis of sarcopenia. Finally, the longitudinal follow-up period was limited to 4-years, potentially impacting the ability to establish causality. Longer follow-up periods with repeated muscle strength and performance measurements are necessary to further elucidate the temporal dynamics between sarcopenia and KOA onset.

## Conclusion

In conclusion, there was an association between sarcopenia or possible sarcopenia and the risk of developing KOA during follow-up. Individuals who newly developed sarcopenia or possible sarcopenia were at a higher risk of developing KOA during the four-year follow-up. Therefore, assessing sarcopenia status during community health checks and in routine clinical practice may help identify populations at higher risk of developing KOA. Furthermore, the dynamic assessment of sarcopenia or possible sarcopenia progression may be helpful in mitigating or delaying KOA onset among middle-aged and older adults.

## Ethics approval and consent to participate

Not applicable.

## Data availability

The datasets analyzed during the current study are available in the open CHARLS databases, http://charls.pku.edu.cn/en.

## Declaration of generative AI and AI-assisted technologies in the manuscript preparation process

During the preparation of this manuscript, the authors only used ChatGPT-5 for language polishing. After using this tool, the authors reviewed and revised all text as necessary and take full responsibility for the content of this publication.

## Authors’ contributions

RH and HT are co-corresponding authors. RH and HT designed the study. XM, ZC, JZ, HT, and RH contributed to the acquisition, analysis, and interpretation of the data. XM, ZC, and JZ drafted the manuscript. HH, WZ, and ZP reviewed the literature. RH and HT revised the manuscript. All authors contributed to the article and approved the submission.

## Funding

No funding was obtained for this study.

## Declaration of competing interest

The authors declare that they have no known competing financial interests or personal relationships that could have appeared to influence the work reported in this paper.

## Appendix Supplementary materials

Image, application 1

Image, application 2
