# Supplementary material for: Association of possible sarcopenia, sarcopenia and knee osteoarthritis among middle-aged and older adults: Evidence from the CHARLS cohort
Source: Clinics (Sao Paulo). 2026 Apr 17;81:100949. doi: 10.1016/j.clinsp.2026.100949 (PMC13098465; doi:10.1016/j.clinsp.2026.100949)
Supplement: Supplementary file 1 [file mmc1.docx]

CLINICS-D-25-01096_Supplementary Material

**Table S1** Comparison of baseline characteristics between included and excluded participants.

| **Variables** | **Excluded (n=9672)** | **Included (n=5018)** | **p-value** |
| --- | --- | --- | --- |
| **Age (years)** | 59.5±10.4 | 58.6±8.6 | <0.001 |
| **Gender, n (%)** |  |  | <0.001 |
| Male | 5040 (52.1) | 2424 (48.3) |  |
| Female | 4632 (47.9) | 2594 (51.7) |  |
| **Sarcopenia, n (%)** | 1184 (20.5) | 874 (17.4) | <0.001 |
| **Possible sarcopenia, n (%)** | 1247 (24.6) | 1133 (22.6) | 0.019 |
| **Residential area, n (%)** |  |  | <0.001 |
| Urban | 4537 (46.9) | 1663 (33.1) |  |
| Rural | 5135 (53.1) | 3355 (66.9) |  |
| **Education, n (%)** |  |  | <0.001 |
| Illiterate | 4054 (41.9) | 2275 (45.3) |  |
| Primary school | 1938 (20.0) | 1171 (23.3) |  |
| Middle school | 2147 (22.2) | 1057 (21.1) |  |
| High school/Vocational high school or above | 1510 (15.6) | 515 (10.3) |  |
| **Ever/Current smoke, n (%)** | 4050 (41.9) | 1985 (39.6) | 0.007 |
| **Ever/Current drink,** **n (%)** | 4132 (42.7) | 2096 (41.8) | 0.268 |
| **BMI (kg/m^2^)** | 23.4±3.9 | 23.5±3.9 | 0.108 |

Note: Continuous variables are presented as mean ± standard deviation and compared using the Student’s *t*-test. Numbers (percentages) were used for categorical variables, and the p-value was calculated using the Chi-Square test. Percentages were calculated using non-missing data. Missing values were excluded from the Chi-Square tests.

BMI, Body Mass Index.

**Table S2** Comparison of baseline characteristics between urban and rural participants.

| **Variables** | **Urban (n=1663)** | **Rural (n=3355)** | **p-value** |
| --- | --- | --- | --- |
| **Age (years)** | 58.3±8.7 | 58.8±8.5 | 0.056 |
| **Gender, n (%)** |  |  | 0.089 |
| Male | 775 (46.6) | 1649 (49.2) |  |
| Female | 888 (53.4) | 1706 (50.8) |  |
| **Sarcopenia, n (%)** | 209 (12.6) | 665 (19.8) | <0.001 |
| **Possible sarcopenia, n (%)** | 380 (22.9) | 753 (22.4) | 0.742 |
| **Education, n (%)** |  |  | <0.001 |
| Illiterate | 571 (34.3) | 1704 (50.7) |  |
| Primary school | 384 (23.1) | 787 (23.5) |  |
| Middle school | 438 (26.3) | 619 (18.5) |  |
| High school/Vocational high school or above | 270 (16.3) | 245 (7.3) |  |
| **Ever/current smoke, n (%)** | 617 (37.1) | 1368 (40.8) | 0.012 |
| **Ever/current drink, n (%)** | 676 (40.6) | 1420 (42.3) | 0.257 |
| **BMI (kg/m^2^)** | 24.2±4.0 | 23.1±3.8 | <0.001 |

Note: Continuous variables are presented as mean ± standard deviation and compared using the Student’s *t*-test. Numbers (percentages) were used for categorical variables, and the p-value was calculated using the Chi-Square test.

BMI, Body Mass Index.

**Table S3** Stratified associations between sarcopenia status and incident KOA by residential setting.

| **Sarcopenia status** | **Urban OR (95% CI)** | **p-value** | **Rural OR (95% CI)** | **p-value** | **p for interaction** |
| --- | --- | --- | --- | --- | --- |
| Possible sarcopenia | 1.138 (0.756, 1.711) | 0.536 | 1.740 (1.359, 2.229) | <0.001 | 0.126 |
| Sarcopenia | 1.246 (0.670, 2.315) | 0.487 | 1.479 (1.055, 2.072) | 0.023 |  |

Note: Fully adjusted logistic regression model. Adjusted for age, gender, rural, body mass index, education, smoking, drinking statuses, dyslipidemia, cancer, lung disease, kidney disease, hepatic disease, digestive disease, asthma, emotional and mental disorders, memory-related disease, arthritis, hypertension, hyperglycemia, heart disease, stroke, White Blood Cell, Platelet, Glucose, Triglyceride, C-Reactive Protein, Cholesterol, High-Density Lipoprotein and Hemoglobin. The Odds Ratio (OR) and 95% Confidence Interval (95% CI) were obtained from multivariate logistic regression analyses. p for interaction was derived from the cross-product term between sarcopenia status and residential setting.
