# Supplementary material for: Association of possible sarcopenia, sarcopenia and knee osteoarthritis among middle-aged and older adults: Evidence from the CHARLS cohort
Source: Clinics (Sao Paulo). 2026 Apr 17;81:100949. doi: 10.1016/j.clinsp.2026.100949 (PMC13098465; doi:10.1016/j.clinsp.2026.100949)
Supplement: Supplementary file 2 [file mmc2.pdf]

CHARLS_wave1_physical examination protocol

5. Walking Speed Test

(1) Required Equipment

Measuring tape, stopwatch, masking tape.

(2) Walking Speed Test

PG002_1: Does the participant meet the eligibility criteria for the walking speed test?

1 Yes → Participant ≥ 60 years → Continue

5 No → Participant < 60 years → Skip to PH001

PG002: "Next, I will assess whether you can comfortably walk a short distance (you may use a cane or other necessary assistive devices). First, I need to confirm whether this test is safe for you. Have you recently undergone surgery, sustained an injury, or experienced any other health condition that might limit your ability to walk?"

Interviewer: Select one option:

1......No apparent limitation → Continue

2......Yes, recent surgery → Test not completed. Please answer the questions in the box below.

3......Yes, injury → Test not completed. Please answer the questions in the box below.

4......Yes, other health condition → Test not completed. Please answer the questions in the box below.

"Now let's find a suitable place for the test. We need an open space of about 4 meters without carpet."

Interviewer: Set up a 2.5-meter line with tape.

"This is the walking course. While you walk along this line, I will measure the time. You will be asked to walk this course twice. During the measurement, I will walk beside you. Now I will demonstrate how the test is performed. When the test starts, please begin walking from the starting point."

Interviewer: Demonstrates the test.

PG003: “Do you understand the procedure and are you willing to participate in this test?”

Interviewer: Select one option:

1......Yes → Continue

5......No → Test not completed. Please answer the questions in the box below.

Program logic:

If PG002 = 1 and PG003 = 1 → Go to QG002

If PG002 = 2, 3, 4, or PG003 = 5 → Go to QG001

(Q000) Based on the above answers.

QG001: Interviewer: Why was the walking speed test not completed? (Multiple choices allowed)

1......Participant considered the test unsafe

2......Interviewer considered the test unsafe

3......Participant refused or was unwilling to perform the test

4......Participant attempted but could not complete the test

5......Participant could not understand the procedure

6......Participant had surgery, injury, or other health reasons preventing walking speed test

7......No suitable place for the test

8......Equipment problem

97......Other (please specify): (QG001_1)

→ Proceed to next test item. Please skip to PH001.

Instruction to participant:

“When you walk at your usual pace along the line, I will measure the time. You will walk the line twice at your normal pace. I will walk beside you during the test. Please stand here with your feet together. When I say ‘Start,’ walk to the end of the tape and stop. Are you ready? Start.”

Interviewer: Record results in the table below. (If the participant attempted but could not perform the test, record 993. If the participant refused, record 999.)

Trial	Walking Time (seconds)
1	(QG002) __. (0.0–999 sec)

[If QG002 = 993 or 999 → answer QG001.]

Repeat measurement instruction:
“Now please walk the same course again, at your usual pace, all the way to the end of the tape. Please stand here with your feet together. When I say ‘Start,’ walk to the end and stop. Are you ready? Start...”
Interviewer: Record results (same rules as above).

Trial	Walking Time (seconds)
2	(QG003) __. (0.0–999 sec)

QG004: Interviewer records type of ground:

1......Vinyl/tile/wood floor

2......Carpet

3......Dirt

4......Cement

5......Don’t know

97......other (please specify): __.(QG004_1)

QG005: Interviewer records walking aid (single choice):

1......None

2......Cane or walking stick

3......Crutch, elbow crutch

4......Walker

97......Other (please specify):__.

QG006: Interviewer records participant compliance during the test (single choice):

1......Fully compliant

2......Not fully compliant due to illness, pain, or other symptoms/discomfort

3......Not fully compliant without clear reason

6. Chair Stand Test

(1) Required Equipment

Chair, stopwatch.

(2) Chair Stand Test

Instruction:

"Now I will ask you to do the following movement. Please cross your arms over your chest. When I say 'Are you ready? Stand up,' stand up straight and sit down again as quickly as possible, repeating this five times without stopping in between and without using your arms for support."

PH001: "Do you understand the procedure and are you willing to participate in this test?"

Interviewer: Select one option:

1......Yes → Continue

2......No → Test not completed. Please answer the questions in the box below.

Program logic:

If PH001 = 1 → Go to QH002

If PH001 = 5 → Go to QH001

Q000: Based on the above answers.

QH001: Interviewer: Why did the participant not complete the chair stand test? (Multiple choices allowed)

1......Participant considered the test unsafe

2......Interviewer considered the test unsafe

3......Participant refused or was unwilling to perform the test

4......Participant attempted but could not complete the test

5......Participant could not understand the procedure

6......Participant unable to stand due to surgery, injury, or other health reason

7......No suitable place for the test

8......Equipment problem

97......Other (please specify):__. (QH001_1)

→ Skip to PI001

QH002: Interviewer: Can the participant complete five consecutive stands from a seated position without arm support at their usual pace? (Select one)

1......Yes → Record the time in seconds, keeping two decimal places. (QH003): __. sec

5......No → (QH004): Record the time participant maintained, to two decimal places: __. sec

993......participant attempted but could not complete

999......Participant refused the test

[If QH002 = 993 or 999 → answer QH001.]

QH005: Number of stands completed: _____ (0–5 times)

QH006: Height of the chair seat from the floor: __. cm (0–999 cm)

QH007: Interviewer: Did the participant use their arms during the test? (Select one)

1......Yes

5......No

8......Don't know

3. Handgrip Strength Test

(1) Required Equipment

Hand dynamometer.

(2) Handgrip Strength Measurement

Instruction:

"Now I will test the maximum grip strength of your hands. Please squeeze the handgrip dynamometer with as much force as possible, hold for a few seconds, and then release. I will measure both your left and right hands separately."

Interviewer: Demonstrate the procedure:

Participant stands, holds the dynamometer, bends the elbow at a right angle, and squeezes firmly for a few seconds.

PC001: "Before the test, I need to ensure it is safe for you. In the past six months, have you had surgery, swelling, inflammation, severe pain, or injury in one or both of your hands?"

Interviewer: Select one option:

1......Yes → Test not completed. Answer the following questions.

2......No → Skip to PC003

PC002: "Which hand (in the past six months) had surgery, swelling, inflammation, severe pain, or injury?"

Interviewer: Select one option:

1......Both hands → Test not completed. Answer the questions in the box below.

2......Left hand only → Continue, but do not test the left hand.

3......Right hand only → Continue, but do not test the right hand.

PC003: "Do you understand the procedure and are you willing to participate in this test?"

Interviewer: Select one option:

1......Yes → Continue

5......No → Test not completed. Answer the questions in the box below.

Program logic:

If both hands injured (PC002 = 1) or participant refuses (PC003 = 5) → Go to QC001.

If no injury (PC001 = 5 and PC003 = 1) → Go to QC002.

If left hand injured (PC001 = 1, PC002 = 2, PC003 = 1) → Answer QC004 and QC006; skip QC003 and QC005.

If right hand injured (PC001 = 1, PC002 = 3, PC003 = 1) → Answer QC003 and QC005; skip QC004 and QC006.

Q000: Based on the answers above.

QC001: Interviewer: Why was the handgrip strength test not completed?

1......Participant considered the test unsafe

2......Interviewer considered the test unsafe

3......Participant refused or was unwilling to complete the test

4......Participant attempted but could not complete the test

5......Participant did not understand the procedure

6......Participant unable to perform due to surgery, swelling, or other health reasons

7......No suitable place to conduct the test

8......Equipment malfunction/problem

97...... Other (please specify): __.(QC001_1)

→ Proceed to the next physical test. Skip to PD001.

QC002: “Which hand do you usually use?”

Interviewer: Select one option:

1......Right hand

2......Left hand

3......Both hands equally

QC003, QC004–QC006: Interviewer: Record the measurement results in the table below.

#	Left Hand	Right Hand
1	(QC003) ___ . ___ (0–100], 993, 999 kg	(QC004) ___ . ___ (0–100], 993, 999 kg
2	(QC005) ___ . ___ (0–100], 993, 999 kg	(QC006) ___ . ___ (0–100], 993, 999 kg

Notes:

If the participant attempted but could not complete the test, record 993.

If the participant refused, record 999.

If QC003 or QC004 = 993 or 999 → Answer QC001.

QC007: Effort level of participant (select one):

1......Participant exerted maximum effort

2......Participant did not exert full effort due to illness, pain, or other symptoms/discomfort

3......Participant did not exert full effort without a clear reason

QC008: Interviewer: Participant's position during the test (select one):

1......Standing

2......Sitting

3......Lying down

QC009: Interviewer: Did the participant's arm rest during the test? (select one):

1......Yes

2......No
